# Biomechanical Investigations of a New Model Graft Attachment to Distal Phalanx in Two-Stage Flexor Tendon Reconstruction

**DOI:** 10.3390/jcm15010141

**Published:** 2025-12-24

**Authors:** Tomasz Mazurek, Krzysztof Żerdzicki, Justyna Napora, Marcin Ceynowa

**Affiliations:** 1 Department of Orthopaedic Surgery, Medical University of Gdansk, ul. Nowe Ogrody, 1–6, 80-803 Gdansk, Poland; 2 Faculty of Civil and Environmental Engineering, Gdansk University of Technology, Gabriela Narutowicza 11/12, 80-233 Gdansk, Poland

**Keywords:** hand surgery, two-stage flexor tendon reconstruction, tensile strength, tendon graft–distal attachment

## Abstract

**Background/Objectives**: In two-stage flexor tendon reconstruction, a biomechanically strong connection between the tendon graft, the motor unit, and the distal phalanx of the finger is essential to enable active rehabilitation after surgery. However, the available literature contains few biomechanical studies concerning the strength of this connection. In this study, we tested a new model of this connection involving suturing the tendon graft to the phalanx using an anchor and to the flexor digitorum profundus stump with a three-level continuous suture (palmaris longus—flexor digitorum profundus—anchor (PL-FDP-A)). **Methods**: For this study, we used eight fingers from patients with injuries that were unsuitable for replantation, as well as eight palmaris longus tendons harvested from cadavers. Eight specimens simulating the PL-FDP-A connections were prepared and tested on a tensile testing machine. The elongation of the specimens under a 20 N load (the minimum for active loading) and the force at rupture were assessed. **Results**: The mean rupture strength was 44.53 N (SD 16.27, min. 16.50, max. 64.60), with elongation at 20 N of 4.28 mm (SD 2.65, min. 1.49, max. 9.14). **Conclusions**: Based on our findings, we recommend the PL-FDP-A connection for use in two-stage flexor tendon reconstruction due to (1) rupture values which significantly exceeded the force required for active rehabilitation, and (2) minimal elongation at 20 N, so that motion transmission was not impaired.

## 1. Introduction

Flexor tendon injuries of the hand are difficult to treat due to their tendency to form scars and adhesions which compromise healing and mobility. The formation of adhesions is particularly significant in the sheath zone known as the “no man’s land” [1].

Despite the use of modern atraumatic surgical solutions, improvements in tendon repair techniques, and the application of tendon grafts and silicone implants, adhesions between the tendon and its surroundings limit tendon excursion and lead to finger contractures [2,3,4]. A particularly challenging problem in tendon surgery arises in secondary repairs which result from absent or inadequate intervention in the primary repair. The key to secondary repair is using biomechanically strong techniques to connect the tendon graft to the distal phalanx, allowing active mobilization [5]. The few studies addressing this issue in the available literature provided the basis for our work [6,7,8,9,10,11,12,13,14,15,16,17,18,19,20].

The aim of this study was to simulate and test point-of-failure mechanisms to examine whether the palmaris longus—flexor digitorum profundus—anchor (PL-FDP-A) tendon reconstruction mechanism would be a stable technique for flexor tendon two-stage reconstruction in terms of the possibility of active rehabilitation and motion translation.

The authors hypothesized that use of this technique would enable the minimum recommended loads required for active rehabilitation to be surpassed, and that the technique would prove stable, though possibly falling short in comparison with techniques using larger tendons.

## 2. Materials and Methods

The study used 8 fingers (second, third, or fourth digits) from young adult individuals (25–40 years old). The thumb and the little finger were excluded from the study due to significant anatomical differences in the flexor tendon system. The fingers were obtained from amputated limbs that were not suitable for replantation due to a low chance of replantation success, the condition of the patient, or the mechanism of amputation. After being cleaned of debris, they were wrapped in moist gauze and frozen immediately following the decision that replantation was not indicated.

The inclusion criterion was good tissue condition without fractures, lacerations, contusions or other pathologies, from the level of the proximal interphalangeal joint (PIP) distally. Fingers from individuals with severe or chronic systemic or local diseases were also excluded. All specimens which met those criteria were included with an intention-to-test protocol. No fingers were replaced or discarded once qualified. Written consent for the use of tissues in the study was obtained from patients, along with approval from the Bioethics Committee of the Medical University of Gdansk (KB/13/2024).

Additionally, we used 8 palmaris longus (PL) tendons harvested from fresh adult cadavers. Tendon retrieval was performed within 48 h postmortem. The tendons were obtained via two ~2 cm incisions on the volar aspect of the forearm. Immediately after harvesting, the tendons were cleaned of muscle and paratenon tissue, wrapped in moist gauze, and frozen. Approval for this procedure was also obtained from the Bioethics Committee.

On the day of testing, the finger specimens were thawed by wrapping them in fresh moist gauze and leaving them at 25 degrees Celsius for 10 h. After thawing, the palmar skin and subcutaneous tissue were removed while the tendon sheath and flexor tendons were preserved. In the distal phalanx, a 2 mm diameter transverse hole was drilled through all structures, allowing a wire to be passed for attachment to the upper grip of the testing machine. All testing was carried out immediately after thawing within a six-hour window from conclusion of the thawing process.

**Figure 1:**
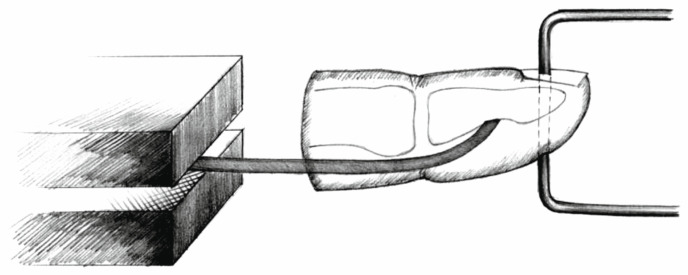
Mechanism of anchoring the tendon graft and the phalangeal bone in the testing machine, and experimental setup, schematic drawing.

**Figure 2:**
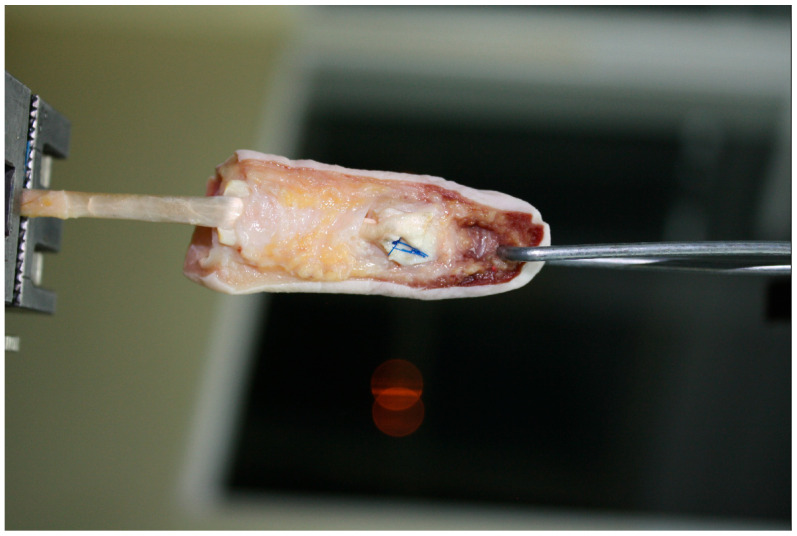
Mechanism of anchoring the tendon graft and the phalangeal bone in the testing machine, and experimental setup, picture.

The PL tendons were thawed in the same manner. An 8 cm segment of each tendon was prepared. The proximal end was reinforced with a 1 cm long braid of 2–0 surgical suture. To this reinforced end, a strip of sandpaper (cloth-based, grit size 180, rough surface facing outward) was attached using cyanoacrylate glue to ensure secure anchorage of the tendon graft in the lower grip of the testing machine, preventing slippage of the specimen (Figure 1 and Figure 2) [21].

**Figure 3:**
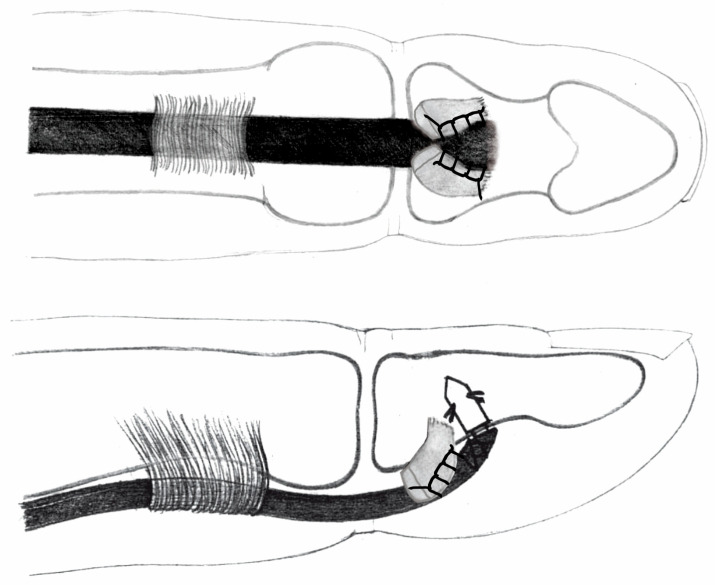
Connection of tendon graft and palmaris longus-PL to the FDP stump with 3-level continuous suture and anchor (PL-FDP-A).

**Figure 4:**
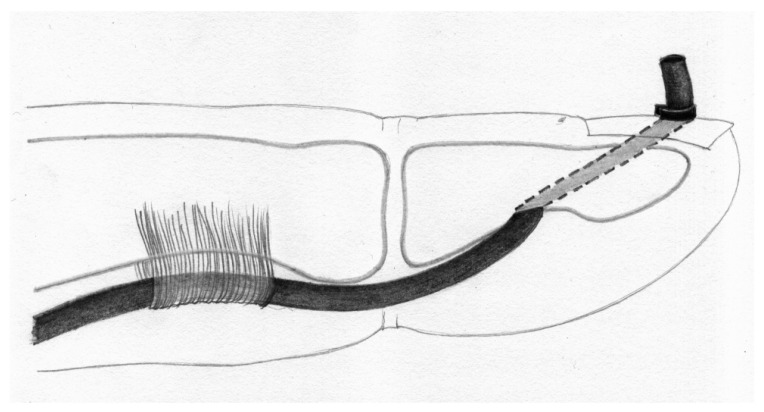
The Snow–Littler technique for pulling out the tendon graft above the nail.

**Figure 5:**
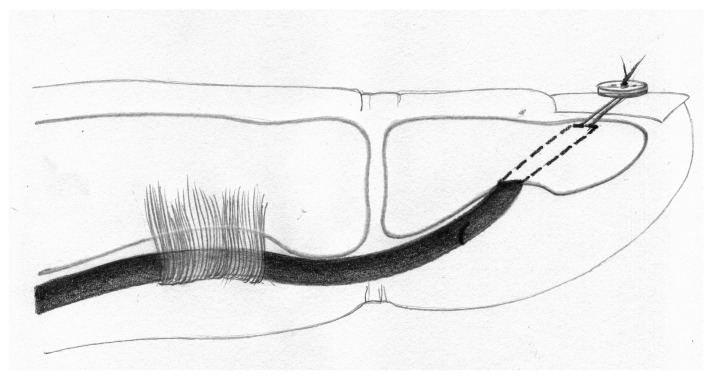
The Wilson technique for pulling the tendon graft into the distal phalanx.

In the prepared finger specimen, through a “window” in the tendon sheath at the site of the flexor digitorum profundus (FDP) tendon insertion into the phalanx, a tenotomy was performed and the FDP was removed, leaving an approximately 1 cm tendon stump attached to the bone. Subsequently, the PL tendon graft was introduced into the tendon sheath canal, and the distal stump of the graft was fixed using a JuggerKnot Soft Anchor One 3-0 Maxbraid (Zimmer Biomet, Warsaw, IN, USA) bone anchor according to manufacturer guidelines on product use, placed at a 90-degree angle to the phalangeal bone surface, with pre-drilling of the cortical bone. The anchor sutures were passed in a triple-layer locking stitch through the bifurcated FDP stump (Figure 3, Figure 4 and Figure 5).

For testing of the mechanical properties of the specimens, we used a Zwick/Roell Z020 universal testing machine (ZwickRoell Sp. z o.o. Sp. K., Wrocław, Poland) equipped with a video extensometer. Two measurement points were marked on each specimen, both of which were continuously tracked by the video extensometer throughout the entire test. The experiments were conducted at room temperature of 25 °C. After clamping the specimens in the machine grips, a preload of 1 N was applied. Subsequently, the specimens were subjected to tensile testing with a loading rate of 5 N/s. During testing, the specimens were kept moist with physiological saline solution.

We evaluated the elongation of each specimen under a load of 20 N as well as the ultimate load at failure. The measurement data were automatically recorded and processed using the TestXpert III V 1.7 (ZwickRoell Sp. z o.o. Sp. K., Wrocław, Poland) software dedicated to the system.

**Figure 6:**
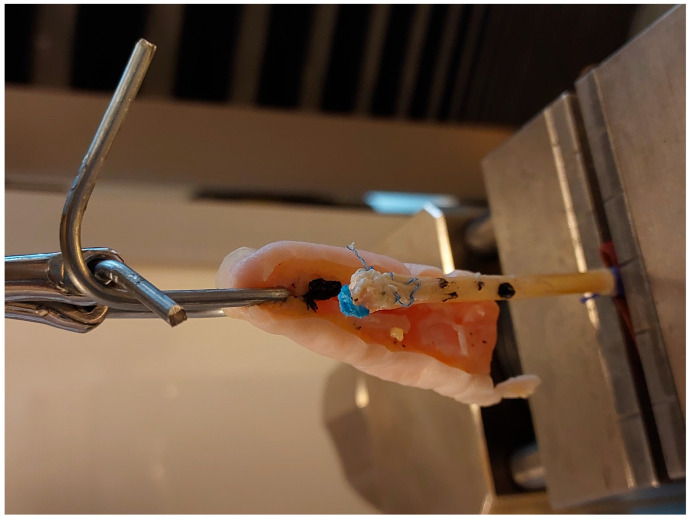
Anchor pull-out, the most common mode of specimen failure in our material.

Three modes of specimen failure were identified:

1. Graft pull-out (suture-induced graft damage).
2. Suture rupture.
3. Anchor pull-out (Figure 6).

## 3. Results

In the tested PL-FDP-A specimens, the mean ultimate failure load was 44.53 N (SD 17.56, min. 16.50, max. 64.60). The mean elongation after applications of 20 N was 4.08 mm (SD 2.63, min. 1.49, max. 9.14). Among the eight specimens, five demonstrated anchor pull-out, two showed suture rupture, and one exhibited graft pull-out. Table 1 demonstrates each specimen in greater detail.

There are differences in all four parameters for each of the failure mechanisms (Table 2); however, the small sample size does not allow for a reliable statistical analysis.

## 4. Discussion

In the history of tendon graft–distal phalanx connections, a variety of surgical techniques have been applied. The first was simple fixation of the tendon graft to the bifurcated FDP stump at the phalanx with two mattress sutures.

Other methods include embedding the graft into the phalangeal bone. Koch in 1944 and Pulvertaft in 1965 described a procedure in which the graft was passed through a transverse tunnel in the phalanx like a lasso [22].

A modification of this method involved anchoring the graft using a suture passed transversely through the phalanx [15]. Bunnell introduced the pull-out method, which involved passing a removable metal suture through the tendon graft–FDP stump junction and exteriorizing it at the fingertip [1]. In 1954, Kyle and Eyre-Brook proposed passing the suture through the phalanx to the nail bed and securing it with a button [23,24].

Both the original Bunnell method and that of Kyle and Eyre-Brook caused compression and necrosis of the soft tissues around the nail bed and fingertip [23]. In 1960, Tubiana used removable sutures passed through an oblique hole in the phalanx and exteriorized at the nail plate with a rolled gauze dressing [25]. Pulvertaft in 1965 applied a technique of bringing the tendon graft out through the fingertip and adjusting its length by clamping it above the skin with a vascular clip [22].

All of the aforementioned techniques involve the disadvantage of soft tissue damage or technical difficulty. In 1971, Snow and Littler proposed passing the graft through an oblique bony canal in the phalanx, exteriorizing it at the nail plate, and stabilizing it at the desired length on the nail plate with a vascular clip (Figure 4) [12].

Although this method offered several advantages, such as anchoring the tendon graft within the bone over a long segment and offering the ability to adjust graft length, it also had drawbacks, including the need to create a wide hole in the phalanx, thereby risking damaging the nail matrix. Considering these limitations, in 1985 Wilson modified this technique by restricting the phalangeal bone hole to the cancellous bone only [26].

This modification reduced damage to the nail plate but eliminated the possibility of adjusting graft length. In 1995, Silfverskiöld and May described a two-stage technique of harvesting the PL graft [23]. In the first stage, the ends of the PL graft were reinforced with Mersilene sutures and then buried within the tissues. After 2–3 months, the graft was retrieved with both reinforced ends surrounded by connective tissue, which allowed for secure reattachment. Morrison and Schlicht harvested the PL with a fragment of the calcaneus [27]. This bony fragment enabled stable anchorage of the tendon graft in the osseous portion of the phalanx.

In parallel with the introduction of anchors for stabilizing the tendon graft to bone, studies began to appear comparing the biomechanical strength of tendon-to-bone fixation using anchors with that of classical techniques [6,28,29].

One of the largest biomechanical studies was conducted by one of the authors [8]; in this work, the biomechanical properties of the most common configurations were compared as follows:

1. Palmaris longus to flexor digitorum profundus (PL-FDP): force at rupture averaged 20.09 N (SD 6.25), elongation after applications of 20 N was 11.61 mm (SD 0.32). Among the eight specimens, seven exhibited graft pull-out, one showed suture rupture.
2. Palmaris longus above the nail (PL-N) (Snow–Littler technique [12]): mean force at rupture was 9.16 N (SD 0.95), elongation after applications of 20 N was impossible in all specimens. Among the eight specimens, all eight exhibited graft pull-out.
3. Palmaris longus to the bone (PL-B) (Wilson technique, Figure 5): force at rupture was 26.23 N (SD 5.81), elongation after applications of 20 N was 11.23 mm (SD 1.74). Among the eight specimens, all eight exhibited suture rupture.
4. Palmaris longus to anchor connection (PL-A): force at rupture was 32.15 N (SD 2.37), elongation after applications of 20 N was 9.34 mm (SD 1.37). Among the eight specimens, all eight exhibited suture rupture.

The aim of this study was to measure specimen elongation under a 20 N load, as well as the load and elongation at failure. The 20 N value is significant in light of the findings of Schuind et al., who demonstrated that it represents the threshold for the safe application of early active motion in tendon graft–phalanx bone connections [4].

The strongest fixation was observed in the PL-A construct, while the weakest was observed in PL-N, the latter being unsuitable for active rehabilitation. In this study, three types of specimen failure were distinguished: graft pull-out, suture rupture, and anchor pull-out. Graft pull-out results from tendon damage caused by sutures and is associated with weak anchorage, as in PL-FDP or PL-N constructs. Suture rupture is linked to mechanically sound constructs where the load is borne solely by the sutures, as in PL-B and PL-A. Notably, no anchor pull-out was observed in the PL-A configuration, a result which is attributable to the high bone density in adults.

A comparable study in which different tendon graft–distal phalanx fixation models were biomechanically evaluated was conducted by Wei et al. [16], who used 36 FDP tendons divided into three groups: a suture button group, a suture anchor group, and a bony attachment group. The best biomechanical outcomes were observed in the latter group, with a failure load of 108.7 N, statistically superior to the pull-out button group (73.5 N) and the suture anchor group (58.2 N). Regarding failure modes, all groups were dominated by suture pull-out from the graft or anchor pull-out, a result which may be related to the advanced age and low bone density of the cadaveric specimens (mean age of 75 years).

The failure loads obtained (58.2–108.7 N), whilst higher than in our study (44.26 N), may be attributed to the use of thicker tendons (FDP) than the PL tendons used in our experiments, which may be a better alternative when performing such reconstructions on patients using autologous tendon transfers. In the study by Skoff et al. [30], no significant difference in failure load was found between the classic Bunnell group and the suture anchor group (approximately 40 N), a finding similar to that reported by Mazurek (approximately 30 N), who compared PL-B and PL-A [8]. Paradoxically, Silva et al. [11] reported that the suture button fixation achieved much better failure loads (58.1 N) than the suture anchor (43.8 N). Brustein et al. [6] compared the use of mini- and micro-anchors and demonstrated superior failure loads for the latter.

Interpretations and comparisons of these studies are limited by differences in the suture material, the suture technique, the testing protocols, and the thickness of the tendon used for fixation [8]. The mode of failure largely depends on bone quality. This is particularly relevant for anchor use: dense, healthy bone in young adults provides the best substrate for anchors, whereas their application is limited in individuals over 75 years of age [7].

A crucial factor for hand function after the second stage of flexor tendon reconstruction is elongation of the construct under a 20 N load, which allows controlled active loading. In our material, this averaged 4.28 mm. The literature lacks biomechanical or clinical data specifically on tendon graft–distal phalanx fixation; however, comparisons can be drawn from flexor tendon repairs in zone II. In a prospective study, Silfversikoid et al. [31] found a mean elongation of 2.6 mm at 3 weeks postoperatively, and demonstrated that this did not affect hand function. In a subsequent study [23] the same author argued that even elongation up to 10 mm does not significantly impair limb function, provided controlled active motion is maintained. In a canine model, Gelberman et al. [32] showed that a gap greater than 3 mm at the repair site does not increase the tendency for adhesions or limit the range of motion. Therefore, our findings suggest that the gapping observed in our model would not impair the range of motion following surgery.

The strength of our study lies in the use of human tissues identical to those used in surgery; this enabled creation of a model very similar to clinical reality (bony phalanx, palmaris longus tendon).

This study is limited by several factors. Firstly, the sample size was relatively small, and due to the difficulty in obtaining appropriate specimens, the rarity of the injury mechanism, and the inclusion criteria for the study, further testing could not be performed. This limited both replicability and obtainment of a sample size sufficiently large for appropriate statistical analysis. In a future study, the data will be expanded to allow for an adequate statistical analysis. Furthermore, cyclic loading tests were not performed. These would have mirrored real-life rehabilitation schemes more closely. The use of cadaveric tissue, where time sensitivity due to tissue decay ought to be considered as a possible source of failure during the experiment, further limited the reliability of results and excluded reliable replicability of tests on one specimen. The study was performed with the assumption that elongation under a 20 N load and ultimate failure load were the key parameters for evaluating the usefulness of this experimental model.

## 5. Conclusions

The proposed PL-FDP-A tendon graft–bony phalanx fixation technique was proven to be a reliable and stable biomechanical solution for two-stage flexor tendon reconstruction, owing to its technical simplicity, material availability, and biomechanical properties. An advantage of this fixation is the use of four sutures for the repair, these being anchored both in the bone by means of the anchor and within the FDP stump itself. Based on our biomechanical findings, the PL-FDP-A construct is recommended as a viable alternative for two-stage flexor tendon reconstruction due to its sufficient failure strength, suitability for early active rehabilitation, and ease of execution.

## Figures and Tables

**Table 1:** Force at failure and tendon extension at different force levels.

Specimen	Maximum Force at Failure (N)	Extension at 20 N (mm)	Mechanism of Failure
1	42.39	5.06	Anchor pullout
2	52.7	1.7	Anchor pullout
3	16.5	3.68	Graft pullout
4	27.6	4.74	Anchor pullout
5	45.1	5.09	Suture rupture
6	64.6	1.49	Anchor pullout
7	62.0	2.71	Anchor pullout
8	43.2	9.14	Suture rupture
Mean	44.53	4.08	
Standard Deviation	17.56	2.63	

**Table 2:** Mean measured values for each failure method.

Mechanism of Failure	Maximum Force at Failure (N)	Extension at 20 N (mm)
Anchor pullout	49.86	3.14
Graft pullout	16.5	3.68
Suture rupture	44.15	7.12

## Data Availability

Further information and raw data is available upon request with the corresponding author.

## References

[1] Bunnell S. (1940). Primary Repair of Severed Tendons the Use of Stainless Steel Wire. Am. J. Surg..

[2] Coyle M.P., Leddy T.P., Leddy J.P. (2002). Staged Flexor Tendon Reconstruction Fingertip to Palm. J. Hand Surg..

[3] Smith P., Jones M., Grobbelaar A. (2004). Two-Stage Grafting of Flexor Tendons: Results after Mobilisation by Controlled Early Active Movement. Scand. J. Plast. Reconstr. Surg. Hand Surg..

[4] Schuind F., Garcia-Elias M., Cooney W.P., An K.N. (1992). Flexor Tendon Forces: In Vivo Measurements. J. Hand Surg. Am..

[5] Boyer M.I., Strickland J.W., Engles D., Sachar K., Leversedge F.J. (2002). Flexor Tendon Repair and Rehabilitation: State of the Art in 2002. J. Bone Jt. Surg..

[6] Brustein M., Pellegrini J., Choueka J., Heminger H., Mass D. (2001). Bone Suture Anchors versus the Pullout Button for Repair of Distal Profundus Tendon Injuries: A Comparison of Strength in Human Cadaveric Hands. J. Hand Surg..

[7] Matsuzaki H., Zaegel M.A., Gelberman R.H., Silva M.J. (2008). Effect of Suture Material and Bone Quality on the Mechanical Properties of Zone I Flexor Tendon-Bone Reattachment with Bone Anchors. J. Hand Surg..

[8] Mazurek T.A. (2011). Secondary repair of flexor tendon injuries in the “no man’s land”—A clinical and experimental study. Ann. Acad. Medicae Gedanensis.

[9] McNally T.A., Hamman J.J., Heminger H., Mass D.P. (2002). The Strength of Distal Fixation of Flexor Digitorum Profundus Tendon Grafts in Human Cadavers. J. Hand Surg..

[10] Moriya K., Yoshizu T., Maki Y. (2020). Flexor Tendon Grafting Using Extrasynovial Tendons Followed by Early Active Mobilization. J. Hand Surg. Glob. Online.

[11] Silva M.J., Hollstien S.B., Brodt M.D., Boyer M.I., Tetro A.M., Gelberman R.H. (1998). Flexor Digitorum Profundus Tendon-to-Bone Repair: An Ex Vivo Biomechanical Analysis of 3 Pullout Suture Techniques. J. Hand Surg. Am..

[12] Snow J.W., Littler J.W. (1971). A Non-Suture Distal Fixation Technique for Tendon Grafts. Plast. Reconstr. Surg..

[13] Sood M.K., Elliot D. (1996). A New Technique of Attachment of Flexor Tendons to the Distal Phalanx without a Button Tie-Over. J. Hand Surg. Eur. Vol..

[14] Tang J.B. (2006). Tendon Injuries across the World: Treatment. Injury.

[15] Viegas S.F. (2006). A New Modification of Two-Stage Flexor Tendon Reconstruction. Tech. Hand Up. Extrem. Surg..

[16] Wei Z., Thoreson A.R., Amadio P.C., An K.N., Zhao C. (2013). Distal Attachment of Flexor Tendon Allograft: A Biomechanical Study of Different Reconstruction Techniques in Human Cadaver Hands. J. Orthop. Res..

[17] Weiner D.L., Hoffman S., Barsky A.J. (1968). Improved Method for Distal Attachment of Flexor Tendon Grafts. Modification of Stenström Technique. Plast. Reconstr. Surg..

[18] Wilson S., Sammut D. (2003). Flexor Tendon Graft Attachment: A Review of Methods and a Newly Modified Tendon Graft Attachment. J. Hand Surg..

[19] Mazurek T., Strankowski M., Ceynowa M., Rocławski M. (2011). Tensile Strength of a Weave Tendon Suture Using Tendons of Different Sizes. Clin. Biomech..

[20] Koch S.L. (1978). Division of the Flexor Tendons within the Digital Sheath. Surg. Gynecol. Obs..

[21] Pulvertaft R.G. (1965). Suture Materials and Tendon Junctions. Am. J. Surg..

[22] Pulvertaft R.G. (1956). Tendon Grafts for Flexor Tendon Injuries in the Fingers and Thumb. J. Bone Jt. Surg..

[23] Silfverskiöld K.L., May E.J. (1995). Early Active Mobilization of Tendon Grafts Using Mesh Reinforced Suture Techniques. J. Hand Surg. Br..

[24] Kyle J.B., Eyre-Brook A.L. (1954). The Surgical Treatment of Flexor Tendon Injuries in the Hand: Results Obtained a Consecutive Series of 57 Cases. Br. J. Surg..

[25] Tubiana R. (1960). Grafts of the flexor tendons of the fingers and of the thumb. Technic and results. Rev. Chir. Orthop. Reparatrice Appar. Mot..

[26] Wilson R.L. (1985). Flexor Tendon Grafting. Hand Clin..

[27] Morrison W.A., Schlicht S.M. (1992). The Plantaris Tendon as a Tendo-Osseous Graft. Part II. Clinical Studies. J. Hand Surg. Br..

[28] Latendresse K., Dona E., Scougall P.J., Schreuder F.B., Puchert E., Walsh W.R. (2005). Cyclic Testing of Pullout Sutures and Micro-Mitek Suture Anchors in Flexor Digitorum Profundus Tendon Distal Fixation. J. Hand Surg. Am..

[29] McCallister W.V., Ambrose H.C., Katolik L.I., Trumble T.E. (2006). Comparison of Pullout Button versus Suture Anchor for Zone I Flexor Tendon Repair. J. Hand Surg. Am..

[30] Skoff H.D., Hecker A.T., Hayes W.C., Sebell-Sklar R., Straughn N. (1995). Bone Suture Anchors in Hand Surgery. J. Hand Surg. Br..

[31] Silfverskiöld K.L., Andersson C.H. (1993). Two New Methods of Tendon Repair: An in Vitro Evaluation of Tensile Strength and Gap Formation. J. Hand Surg. Am..

[32] Gelberman R.H., Woo S.L.Y., Lothringer K., Akeson W.H., Amiel D. (1982). Effects of Early Intermittent Passive Mobilization on Healing Canine Flexor Tendons. J. Hand Surg. Am..

